# Cognitive Abilities in Malawi Cichlids (*Pseudotropheus* sp.): Matching-to-Sample and Image/Mirror-Image Discriminations

**DOI:** 10.1371/journal.pone.0057363

**Published:** 2013-02-20

**Authors:** Stefanie Gierszewski, Horst Bleckmann, Vera Schluessel

**Affiliations:** Institute of Zoology, Rheinische-Friedrich-Wilhelms Universität Bonn, Bonn, Nordrhein-Westfalen, Germany; Lund University, Sweden

## Abstract

The ability to recognize and distinguish between visual stimuli is fundamental for everyday survival of many species. While diverse aspects of cognition, including complex visual discrimination tasks were previously successfully assessed in fish, it remains unknown if fish can learn a matching-to-sample concept using geometrical shapes and discriminate between images and their mirror-image counterparts. For this purpose a total of nine Malawi cichlids (*Pseudotropheus* sp.) were trained in two matching-to-sample (MTS) and three two-choice discrimination tasks using geometrical, two-dimensional visual stimuli. Two out of the three discrimination experiments focused on the ability to discriminate between images and their mirror-images, the last was a general discrimination test. All fish showed quick associative learning but were unable to perform successfully in a simultaneous MTS procedure within a period of 40 sessions. Three out of eight fish learned to distinguish between an image and its mirror-image when reflected vertically; however none of the fish mastered the task when the stimulus was reflected horizontally. These results suggest a better discrimination ability of vertical compared to horizontal mirror-images, an observation that is widespread in literature on mirror-image discrimination in animals. All fish performed well in the general visual discrimination task, thereby supporting previous results obtained for this species.

## Introduction

According to Shettleworth [1] *“cognition refers to the mechanisms by which animals acquire, process, store, and act on information from the environment*”. Cognitive abilities play an important role in foraging, mate-choice, predator avoidance, navigation and orientation [2]. For these behaviors, perception of environmental stimuli through the animal's sensory systems is essential [3], which should be tuned to the animal's specific needs and its respective habitat. It can be assumed that the higher an animal's cognitive abilities are developed the more flexible it may react in response to changing environments or challenging situations. Numerous experiments have been conducted on a variety of species, and simple to complex discrimination abilities have been found in both invertebrates [cephalopods [4]; crustaceans [5]; insects: ants [6], bees [7]] as well as vertebrates (fish [8]; amphibians [9]; reptiles [10], [11]; birds [12], [13]; mammals: monkeys [14], rats [15]).

### Visual form discrimination abilities in fish

The first experimental study that provided evidence of visual form discrimination in fish was conducted in 1926 by Schaller [16] who trained Eurasian minnows (*Phoxinus phoxinus*) to discriminate between different colors and geometrical shapes. In the 1950s, Herter and co-workers (for a review see [17]) continued on from this work by training several fish species in a wide range of visual discrimination tasks, many of which served as models for the studies that followed. For example, discrimination of color and line orientation was successfully tested in zebrafish (*Danio rerio* [8], [18]) and weakly electric elephantnose fish (*Gnathonemus petersii*) successfully learned to distinguish geometrical shapes [19]. Two- and also three-dimensional form discriminations were performed by Ambon damselfish (*Pomacentrus amboinensis* [20]). Newer studies on distance discrimination were performed on goldfish (*Carassius auratus auratus*) by Frech et al. [21] using stimuli differing in spatial depth. Several more recent studies also focused on subjective and illusory contours [22], [23] and the ability to recognize objects that were partly occluded [24]. Various form discrimination abilities in cichlids have already been tested by Herter [17], Behrend and Bitterman [25], Mark [26], Mark and Maxwell [27] and Schluessel et al. [28]. The study by Schluessel et al. [28] furthermore demonstrated that *Pseudotropheus* sp. is able to form object categories, i.e. ‘fish’ vs. ‘snail’.

### The discrimination of an image and its mirror-image

With regard to form discrimination abilities, many studies in non-fish species have concentrated on image/mirror-image stimulus pairs (for a review see [29]). One stimulus is shown in its normal orientation while the second stimulus (identical to the first) is reflected along its vertical or horizontal axis. It is then tested whether the subject can discriminate between the two presented stimuli, differing only in orientation. Most studies refer to early work by Sutherland [4], [30], [31], who investigated the visual discrimination abilities in the invertebrate *Octopus vulgaris*. He showed that octopus could readily discriminate between a vertical and a horizontal rectangle ( | vs.—), but could not distinguish between oblique rectangles (/vs.\). Furthermore it was shown that octopus could readily discriminate between a U-shape and its vertical mirror-image (rotated 180°), but had difficulties distinguishing between a ‘lying’ U-shape (rotated 90° to the left or right) and its horizontal mirror-image. Similar findings had previously been shown in rats [32]. Additional studies were performed to test whether these findings also applied to other animals or even humans. Rudel and Teuber [33] discovered similar difficulties in 3–4 year old children which were confirmed by Bornstein et al. [34] in human infants and by Corballis and McLaren [35] in 20–25 year old men and women. Additionally, experiments using pigeons (*Columba livia* [36]), cats [37], and rhesus monkeys (*Macaca mulatta* [38]) revealed difficulties in discrimination between horizontal image/mirror-image pairs in contrast to vertical mirror-images. Furthermore, a general problem concerning discrimination of mirror-image stimuli compared to non-mirror-image stimuli was discovered in various species [36], [38]–[43]. However, there were also species that showed no limitation in discriminating between image/mirror-image stimuli, such as baboons (*Papio papio* [44]), lion-tailed macaques (*Macaca silenus* [45]), sea lions (*Zalophus californianus* [46]), pigeons (*Columba livia* [41]), and bumblebees (*Bombus impatiens* [47], [48]). The only study on mirror-image discriminations in fish was conducted by Mackintosh and Sutherland [49] who tested goldfish (*Carassius auratus auratus*) in discriminations of rectangles differing in orientation. Goldfish needed fewer training trials to successfully discriminate between vertical and horizontal rectangles ( | vs. —) than when using oblique rectangles (/vs.\). No study has tested image/mirror-image discriminations using symbols other than rectangles in any species of fish.

### Matching-to-sample (MTS)

The first MTS procedures were reported by Blough [50] and Konorski [51] as a technique for investigating short-term memory and discrimination abilities and have since been tested on a variety of species and with various stimuli. In typical MTS procedures, a given sample stimulus has to be chosen out of two subsequent comparison stimuli. The comparison stimuli represent a target stimulus (positive) that is the same as the sample, and a non-corresponding distracting stimulus (negative). Choosing the target stimulus is positively reinforced. Stimuli can be presented in either a simultaneous or delayed MTS procedure. In a simultaneous MTS procedure, the sample stimulus is first shown alone followed by the appearance of the target stimulus and the distracting stimulus on either side. The sample stimulus remains visible in the center of the two comparison stimuli. In the delayed MTS, the sample stimulus is first shown alone and then followed by a delay of a defined period of time. After the delay, only the two comparison stimuli are shown. The subject then has to remember the original sample to make a correct choice. A correct response during a MTS task depends on the identity of the two stimuli (the sample and the distracting stimulus) [52]–[55]. Thus, solving an MTS task requires concept learning, which means the understanding of the concepts of ‘same’ and ‘different’, or at least rule learning [56]–[63]. In concept learning the relationship between stimuli is assessed (stimuli are ‘same’ or ‘different’) whereas in rule learning stimulus specifics (color, size, orientation, etc.) are compared and formed into a rule or guideline that is learned by the subject (‘if the sample is red, then choose red out of the comparison stimuli’). A learned concept can be easily applied to novel stimuli, whereas a rule is typically linked to one set of stimuli. On the basis of the just mentioned points, the use of MTS procedures in experiments makes it possible to investigate ‘higher’ cognitive abilities in the tested subjects. Due to the high abundance of studies using MTS procedures, only a few are mentioned here. Discrimination of auditory stimuli has been tested on tufted capuchin monkeys (*Cebus apella* [64]) as well as on the common bottlenose dolphin (*Tursiops truncatus* [65]). Olfactory stimuli were successfully discriminated by dogs [66] and honeybees (*Apis mellifera* [7]). Numerous studies concentrated on MTS tasks using visual stimuli such as colored or blinking lights (pigeons [50], [52], hens [67], rats [68]), two-dimensional form and color stimuli (pigeons [69], monkeys [57]), and digital computer-drawn color pictures (pigeons [61]). Also, three-dimensional objects (e.g. teapot, soccer ball, plastic toys) were used as visual stimuli in a MTS study using a California sea lion (*Zalophus californianus* [70]). Despite the high number of MTS studies overall, the literature reveals only two examples of MTS testing in fish. The first evidence for the successful use of a MTS procedure in fish was given by Goldman and Shapiro [53] who trained goldfish (*Carassius auratus auratus*) in both identity and oddity matching using colored lights that were presented in a modified three-key chamber. Zerbolio and Royalty [71] also used the MTS paradigm to train goldfish on a color (lights) identity and oddity matching in an avoidance shuttlebox. No study has tested MTS procedures on fish using various shapes and forms.

### Aim of the study

This study was conducted to investigate aspects of the cognitive abilities of the teleost *Pseudotropheus* sp. It was tested if individuals could successfully perform in a simultaneous MTS procedure as well as in advanced visual discrimination tasks using two dimensional shapes and their mirror-images. It was hypothesized that *Pseudotropheus* sp. can learn a simultaneous MTS procedure, as a similar procedure had already been successfully tested in goldfish using color stimuli [53]. Image/mirror-image discriminations had never been tested in a cichlid before. It was assumed though, that abilities would be similar to those of other animals.

## Materials and Methods

### Ethics statement

The research reported herein was performed under the guidelines established by the current German animal protection law. The current study did not include invasive experiments but simple behavioral observations. Therefore no specific permits were needed.

### Experimental animals

Nine *Pseudotropheus* sp. (Teleostei: Cichlidae) ranging from about 4.9 cm to 6.5 cm in standard length were trained. Five fish had already been used in a previous study on visual discrimination and categorization abilities [28]. Four fish were experimentally naïve. A maximum of eight individuals could be housed and was therefore tested at once. Each fish’s gender was determined on the basis of visible egg-spots on the anal fin [72], [73] resulting in seven males and two females. *Pseudotropheus* sp. is a rock-dwelling cichlid, endemic to the shallow waters of Lake Malawi in East Africa. It is a diurnal mouth-breeder, feeding predominantly on algae. Males are highly territorial [72], [74]. The spatial structure of the natural habitats of *Pseudotropheus* sp. is complex with good visibility [74], [75]. Former studies comparing brain structure, brain size, and environmental factors in African cichlids [74]–[78], revealed that species living in complex habitats feature a larger telencephalon, which is considered important for learning and social behavior [79]. African cichlids and especially inhabitants of complex environments like *Pseudotropheus* sp., can be considered well suited experimental subjects for investigating cognitive skills [75], [80].

### Maintenance of the experimental animals

**Figure 1:**
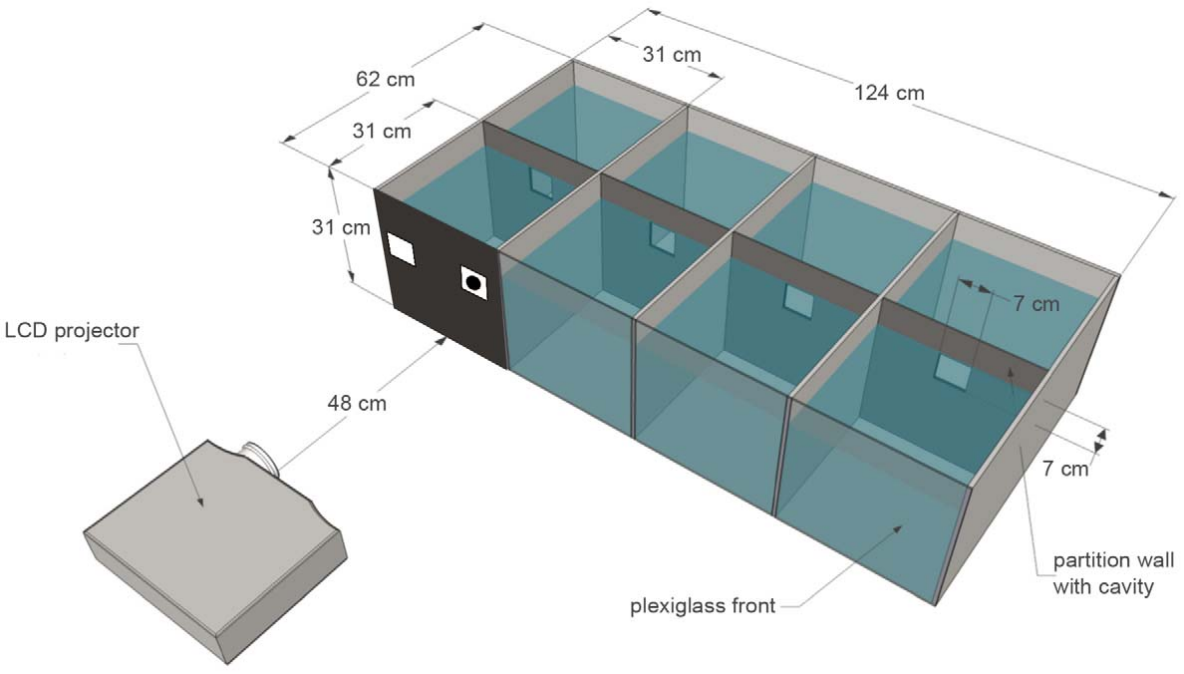
Oblique view of a block of aquaria. One of two blocks of four identical tanks divided by partition walls. The LCD projector used during the experiments was positioned in front of the plexiglass and could be moved sideways. The entire front wall of a tank was used for the projections (indicated in black).

**Figure 2:**
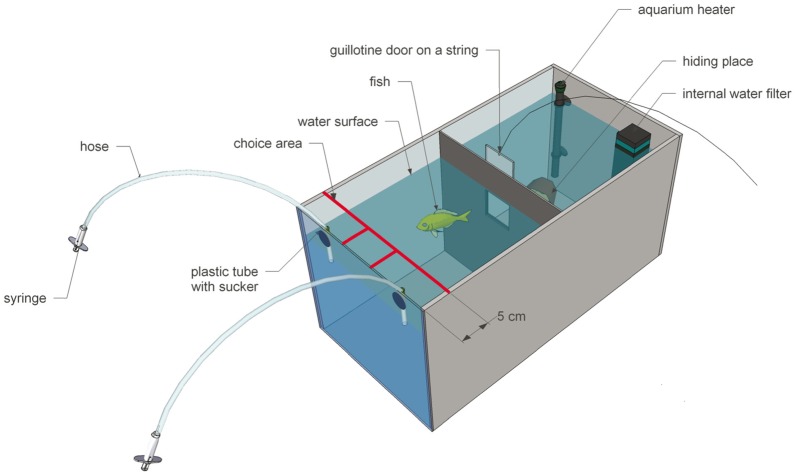
Oblique view of a single tank. Two compartments, separated by a partition wall with a small guillotine door. The experimental area included two feeding apparatuses. Red lines indicate the choice areas. The living area contained a water filter, aquarium heater, and a hiding place. The projection area was below the feeding apparatuses.

Fish were individually kept in tanks (31cm x 31cm x 62cm) made of grey plastic except for a semi translucent, milk-colored plexiglass front (Fig. 1). Tanks were divided into two compartments by a partition (Fig. 1). The back compartment served exclusively as living area, containing an internal water filter (DUETTO COBRA DJC 50, Aquarium Systems Newa, France; 50 liter, 250 l/h), an automatic aquarium heater (HT 50 Tetra®, Germany) and a hiding place. The front compartment, featuring the milk-colored plexiglass front (needed for the experimental projections), also served as the experimental area and included two feeding apparatuses: on each side a flexible tube (Ø 4/6 mm) containing a hose with the reward on one end and a syringe on the other, was fixed in position on the inside wall (Fig. 2). Individuals could access both compartments by swimming through a small guillotine door located in the center of the partition wall. Prior to an experiment the door was closed and only opened at the beginning of each trial. Food (granugreen sera®, Germany) was given exclusively during experiments as a reward after each trial. Aquarium water (approximately 44 liter) was enriched using multivitamins (Atvitol JBL, Germany) and kept at a temperature of 25± 1 °C with a pH-value of 8, and a kH-value of 5. Every few weeks, about a quarter of the water was replaced with fresh water.

### Two-dimensional stimuli used in this study

**Figure 3:**
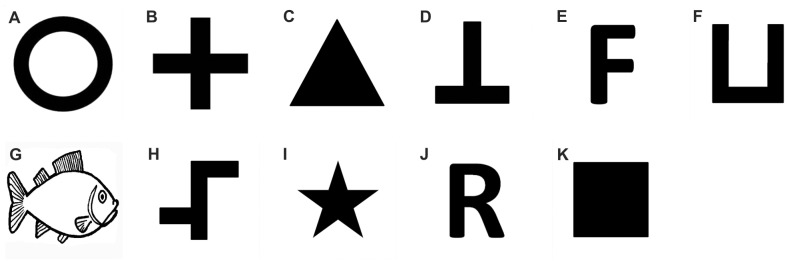
Visual two-dimensional stimuli used in this study. (A) open circle, (B) cross, (C) filled triangle, (D) reversed letter ‘T’, (E) F-shaped form, (F) U-shaped form, (G) line drawing of a fish, (H) asymmetrical shape, (I) star, (J) letter ‘R’, (K) filled square.

As former results showed that *Pseudotropheus* sp. can discriminate between two-dimensional geometrical shapes and line drawings [28], shapes were used (instead of colored lights as in [53]), to test the MTS hypothesis. Stimuli were designed (CorelDRAW® X4; see Fig. 3) and presented (Microsoft® PowerPoint 2010) as black shapes on a light grey background. Four different PowerPoint presentations (A-D, see Tables 1–3) were created for each stimulus pair, containing different predetermined sequences for automatic presentation during experiments. Presentations were used one after another to prevent individuals from memorizing recurring combinations of stimuli and anticipating the side the positive stimulus would be shown on in the next trial. Within presentations, stimuli were arranged using pseudorandom tables as described in [81] and shown equally often on either side (left and right). The same stimulus was never shown more than twice in a row on the same side. These precautions were taken to prevent any side biases. Stimuli were projected onto the whole plexiglass front of the tank (Fig. 1) using an LCD projector (EMP-54 EPSON®, Germany; settings: brightness 5, contrast -10) which was located directly in front of the tank and connected to a notebook (Samsung R60*^plus^*). Markings on the plexiglass guaranteed a projection onto the same fixed spot in each session. Stimuli were shown in stimulus fields measuring 7×7 cm, which were presented on a black background to prevent fish from being totally blinded by the entrance of too much light (Fig. 1). The projection of stimulus fields was positioned at the height-level of the guillotine door to ensure that individuals could immediately see the stimuli when approaching (in case of a transparent door during MTS procedures) or exiting (in case of an opaque door during two-choice discriminations) the guillotine door (Fig. 1).

**Table 1:** Stimulus sequences used in experiment 1a.

	A		B		C		D	
Trial	P	N	P	N	P	N	P	N
**1**	+ R	o	+ L	o	o L	+	o R	+
**2**	+ L	o	+ L	o	+ R	o	+ L	o
**3**	o R	+	o R	+	o L	+	o L	+
**4**	+ R	o	o L	+	+ R	o	+ R	o
**5**	o L	+	+ R	o	+ L	o	o R	+
**6**	o R	+	o L	+	o R	+	o L	+
**7**	+ L	o	o R	+	+ R	o	+ R	o
**8**	o R	+	+ L	o	+ L	o	+ L	o
**9**	+ L	o	+ R	o	o R	+	o R	+
**10**	o L	+	o R	+	o L	+	+ L	o

The composition of four different predetermined sequences A-D (PowerPoint presentations), containing 10 trials each, used in experiment 1a. For each trial, the positive stimulus that served as sample and corresponding stimulus and its position are shown. The negative stimulus that served as distracting stimulus is also shown for each trial. P =  positive stimulus; N =  negative stimulus; + =  cross; o =  circle; R =  right; L =  left.

**Table 2:** Stimulus sequences used in each step of experiment 1b.

Step 1								
	A		B		C		D	
Trial	P	N	P	N	P	N	P	N
1	+ R	□	+ L	□	o L	□	o R	□
2	+ L	□	+ L	□	+ R	□	+ L	□
3	o R	□	o R	□	o L	□	o L	□
4	+ R	□	o L	□	+ R	□	+ R	□
5	o L	□	+ R	□	+ L	□	o R	□
6	o R	□	o L	□	o R	□	o L	□
7	+ L	□	o R	□	+ R	□	+ R	□
8	o R	□	+ L	□	+ L	□	+ L	□
9	+ L	□	+ R	□	o R	□	o R	□
10	o L	□	o R	□	o L	□	+ L	□

Step 2								
	A		B		C		D	
Trial	P	N	P	N	P	N	P	N
1	+ R	□	▵ L	□	o L	□	▵ R	□
2	+ L	□	+ L	□	+ R	□	+ L	□
3	o R	□	o R	□	T L	□	T L	□
4	▵ R	□	T L	□	▵ R	□	+ R	□
5	▵ L	□	+ L	□	+ L	□	o R	□
6	o R	□	▵ R	□	o R	□	o L	□
7	T L	□	o R	□	T R	□	T R	□
8	T R	□	+ L	□	+ L	□	+ L	□
9	+ L	□	T R	□	o R	□	o R	□
10	o L	□	o R	□	▵ L	□	▵ L	□

Step 3								
	A		B		C		D	
Trial	P	N	P	N	P	N	P	N
1	▵ R	□	▵ L	□	T L	□	T R	□
2	▵ L	□	▵ L	□	▵ R	□	▵ L	□
3	T R	□	T R	□	T L	□	T L	□
4	▵ R	□	T L	□	▵ R	□	▵ R	□
5	T L	□	▵ L	□	▵ L	□	T R	□
6	T R	□	T R	□	T R	□	T L	□
7	▵ L	□	T R	□	▵ R	□	▵ R	□
8	T R	□	▵ L	□	▵ L	□	▵ L	□
9	▵ L	□	▵ R	□	T R	□	T R	□
10	T L	□	T R	□	T L	□	▵ L	□

Step 4								
	A		B		C		D	
Trial	P	N	P	N	P	N	P	N
1	▵ R	T	▵ L	T	T L	▵	T R	▵
2	▵ L	T	▵ L	T	▵ R	T	▵ L	T
3	T R	▵	T R	▵	T L	▵	T L	▵
4	▵ R	T	T L	▵	▵ R	T	▵ R	T
5	T L	▵	▵ L	T	▵ L	T	T R	▵
6	T R	▵	T R	▵	T R	▵	T L	▵
7	▵ L	T	T R	▵	▵ R	T	▵ R	T
8	T R	▵	▵ L	T	▵ L	T	▵ L	T
9	▵ L	T	▵ R	T	T R	▵	T R	▵
10	T L	▵	T R	▵	T L	▵	▵ L	T

The composition of four different predetermined sequences A-D (PowerPoint presentations), containing 10 trials each, used in steps 1–4 of experiment 1b. For each trial, the positive stimulus that served as sample and corresponding stimulus and its position are shown. The negative stimulus that served as distracting stimulus is also shown for each trial. P =  positive stimulus; N =  negative stimulus; + =  cross; o =  circle; ▵ =  triangle; T =  reversed letter ‘T’; □ =  blank square; R =  right; L =  left.

**Table 3:** Stimulus sequences used in experiments 2 and 3.

Trial	A	B	C	D
1	R	L	R	L
2	R	R	L	R
3	L	L	R	L
4	R	R	L	L
5	L	R	R	R
6	L	L	L	R
7	R	L	R	L
8	L	R	R	L
9	R	L	L	R
10	R	L	R	L

The composition of four different predetermined sequences A-D (PowerPoint presentations), containing 10 trials each, used in experiments 2 and 3. For each trial, the position of the positive stimulus is shown. The sequences remained the same for every tested stimulus. R =  right; L =  left.

### Experimental procedure

Experiments were conducted six days a week. There were two sessions per day à ten trials. Every trial was followed by an intertrial interval (ITI) of one minute. Sessions took place in the morning and in the afternoon, resulting in a food deprivation period of about 5 hours between the 1^st^ and the 2^nd^ session and an overnight deprivation period of about 18 hours. During trials, both feeding apparatuses were filled with the same amount of food (granugreen sera®, Germany; one pellet each) to prevent subjects from using uncontrolled olfactory or visual clues. Food pellets were soaked in water and then inserted into the feeding hose (Fig. 2). To prevent pellets from dropping out randomly, a small amount of air was soaked in after the pellet. The learning criterion for all experiments was set to achieving at least 70 % correct choices in three subsequent sessions. The probability of achieving the criterion by chance was less than 5 % (χ^2^-test, p<0.05). Entering the ‘choice area’ (see Fig. 2) in front of the positive stimulus was recorded as a correct choice and the fish was rewarded with food by manually releasing the food pellet out of the feeding apparatus. Afterwards, the individual was moved to the rear compartment and the ITI began. In case of an incorrect choice, the stimulus presentation was turned off, no reward was given and the individual was moved to the rear compartment where the ITI started. Only first choices were counted. Reaction time, i.e. the time the individual needed to pass through the door and enter the ‘choice area’, was measured with an accuracy of 1/100 s for each trial. Choice (left or right) was also recorded per trial. Every session was observed and filmed using a webcam (Webcam C210 Logitech®, Switzerland; 1.3MP, 640×480 pixel video resolution, 30 fps) situated above the experimental compartment of the tank. This way, the experiments were conducted without any visible presence of the experimenter, preventing an influence on the fish’s choice by indirectly giving clues. To provide a constant illumination of the tanks during the presentation of the stimuli, the window in the experimental room was covered with an opaque curtain. The room was then illuminated by neon tubes (SYLVANIA Luxline Plus, F58W/840-TS, Cool White deluxe, Germany) that were not located directly above the aquaria but on the other side of the room.

### Pre-training for habituation to the experimental procedure

The purpose of pre-training was to habituate the naïve subjects to the experimental procedure. At this stage, fish were positioned in the back compartment and trained to swim through the door to the front of the aquarium to receive a reward out of two possible feeding apparatuses. During pre-training, subjects were rewarded equally often on the left and on the right side to avoid the development of side biases. Fish were never rewarded for choosing ‘the middle’. In a next step, subjects were habituated to the guillotine door and to the presentation of the beamer lights by using a PowerPoint presentation containing three light grey squares (7×7 cm) on a black background. These squares were also used during the experiments, where they featured the test stimuli. Once the fish had learned to swim reliably to the front compartment and obtain food from the feeding apparatuses, training started. ‘Swim reliably’ was defined as swimming through the door without hesitation. Habituated and trained individuals normally reached the front wall within <2 seconds.

### Experiments

The number of sessions per experiment was limited to 35–40, because former studies [20], [22], [28] revealed that learning success in fish usually occurred within this period. A short overview of the experiments is given below in Table 4.

**Table 4:** Overview of the different MTS and two-choice discrimination experiments.

Experiment 1	Matching-to-sample procedure
	1a – MTS (simultaneous)
	1b – MTS (simultaneous); divided into four steps for simplification
Experiment 2	Two-choice discrimination
	2a – image/mirror-image stimulus pairs
	F-shape (horizontal mirror-image)
	F-shape (vertical mirror-image)
	U-shape (vertical mirror-image)
	‘fish’-shape (horizontal mirror-image)
	2b - distinct geometrical stimulus pairs; mirror-image transfer tests
	asymmetrical shape vs. star
	asymmetrical shape vs. ‘R’ with transfer tests (T1 and T2)
	T1: vertical mirror-image of stimulus pair
	T2: horizontal mirror-image of stimulus pair
Experiment 3	Two-choice discrimination
	filled square vs. blank stimulus field

Short information is given concerning the performed experiments and therein used methods and stimuli.

### Experiment 1a: simultaneous MTS of ‘cross’ and ‘circle’

**Figure 4:**
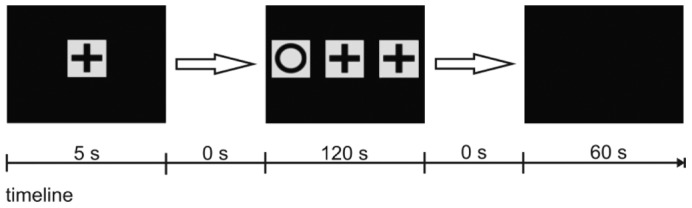
Example of a sequence of PowerPoint slides used in a trial of experiment 1a. The figure shows the chronology of a possible sequence used in a trial of experiment 1a, consisting of three slides presenting the stimuli in a simultaneous MTS. The timeline indicates the duration of each slide's presentation in seconds. The last slide denotes the ITI.

In experiment 1a, discrimination of a cross vs. a circle was tested. The successful use of two stimuli in MTS testing was shown in different studies [7], [50], [56], [67], [68], [82]. The sample stimulus was first shown on its own for 5 seconds and then remained in the center of the screen while a test stimulus (one ‘target’ or positive stimulus and one ‘distracting’ or negative stimulus) appeared on either side (Fig. 4 and Table 1). Fish were given two minutes to choose between the two test stimuli. The transparent guillotine door was used to enable the fish to watch the presentation of the sample stimulus prior to opening the door. The door was opened once the target as well as the distracting stimulus were presented on the right and left of the plexiglass front. To pass this experiment successfully, it was necessary for the individual to stay at the door, watch the presentation of the sample stimulus and, after the guillotine door was opened, swim to the positive stimulus.

### Experiment 1b: simultaneous MTS ‘step by step’

**Figure 5:**
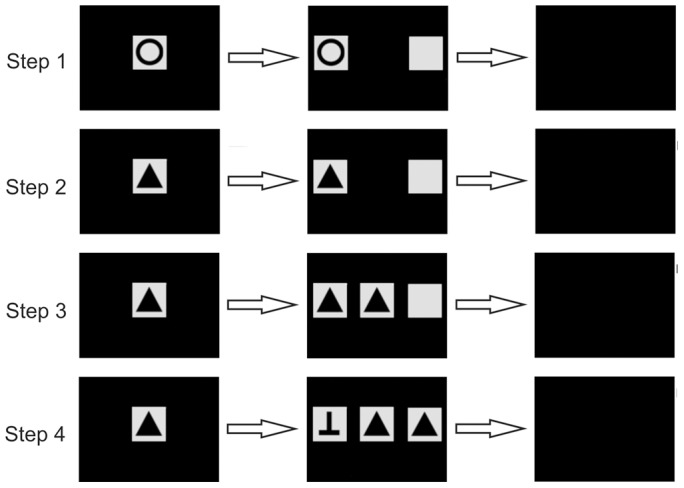
Examples of the sequences of PowerPoint slides used in the different steps of experiment 1b. For every step (1–4) of the experiment, possible slides presenting the stimuli and their duration per trial are given. In steps 1–3, a blank square instead of a distracting stimulus is used. The last slide denotes the ITI.

Because experiment 1a did not yield successful results, experiment 1b was designed to teach the MTS procedure in small steps with increasing complexity. The general presentation of stimuli remained the same as in experiment 1a but stimuli composition and the number of the used stimuli was varied. There were four successive steps:

Step 1: Only one of the stimuli (‘cross’ or ‘circle’) was shown alternately on the left or right side of the screen, without another stimulus for comparison. The remaining square was blank. The sample stimulus was shown for 5 seconds in the center of the presentation and then disappeared. Afterwards, the individuals had two minutes to choose between the corresponding stimulus (positive) and an empty field (negative; Fig. 5, step 1; Table 2).

Step 2: After reaching the learning criterion in step 1, two new stimuli (black triangle and the reversed letter ‘T’) were introduced, so that now a total of four different stimuli were presented to the individuals. During each session, cross and circle were both shown in three trials each, and triangle and reversed ‘T’ were both shown in two trials each (out of ten trials in total; Fig. 5, step 2; Table 2). As in step 1, an empty field served as the negative stimulus.

Step 3: After reaching the learning criterion in step 2, the fish were introduced to a simultaneous MTS showing only the new stimuli (black triangle and reversed ‘T’). The sample stimulus remained visible in the center of the presentation and individuals had to choose between the corresponding stimulus (positive) and an empty field (negative). After reaching the learning criterion, the individuals had to perform several more sessions to check whether their performance remained steady above the 70 % threshold (Fig. 5, step 3; Table 2).

Step 4: After reaching the learning criterion in step 3, experiment 1a was repeated by conducting a simultaneous MTS using the black triangle and the reversed ‘T’ as stimuli (Fig. 5, step 4; Table 2).

### Experiment 2a: image/mirror-image discrimination

**Figure 6:**
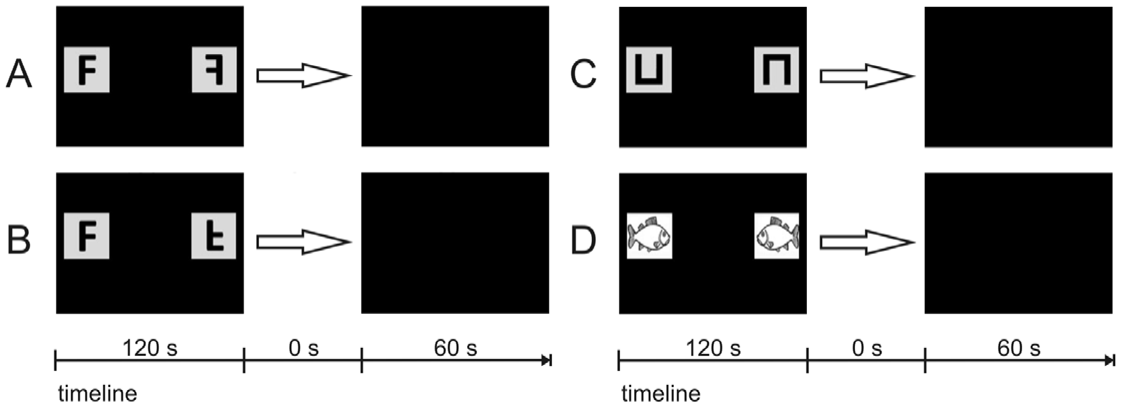
Chronology of the sequences of PowerPoint slides used in the different steps of experiment 2a. The positive stimulus is shown on the left. The timeline indicates the duration of each slide's presentation in seconds. The last slide denotes the ITI. (A) The F-shape and its horizontal mirror-image. (B) The F-shape and its vertical mirror-image. (C) The U-shape and its vertical mirror-image. (D) The ‘fish’ stimulus and its horizontal mirror-image.

In experiment 2a, fish had to perform a two-choice image/mirror-image discrimination task. In four different tests (A-D), individuals were given pairs of image/mirror-image stimuli and were trained to choose the non-reflected image to get a reward. Most of the used stimuli were deliberately chosen to provide a certain comparability to existing data of former studies on image/mirror-image discrimination. In the following, the stimuli that were used will be presented in chronological order (also see Table 3):

Tests A and B: F-shape (horizontal and vertical mirror-image) as used in [36], [38]. Image and mirror-image of F-shapes could not be matched by rotating the shape but only by reflecting it along the vertical or horizontal axis (Fig. 6 A and Fig. 6 B).

Test C: U-shape (vertical mirror-image) as used in [36], [37], [83] (see Fig. 6 C). Image and mirror-image of U-shapes could be matched by either rotation (180°) or reflection along the horizontal axis. Therefore, fish could solve the task in two possible ways.

Test D: Drawing of a fish and its horizontal mirror-image. The picture of the fish looking to the right was rewarded (Fig. 6 D). Image and mirror-image of the fish drawing could only be matched by reflection along its vertical axis. Only four individuals performed on this last test.

### Experiment 2b: discrimination of distinct stimulus pairs with mirror-image transfer tests

**Figure 7:**
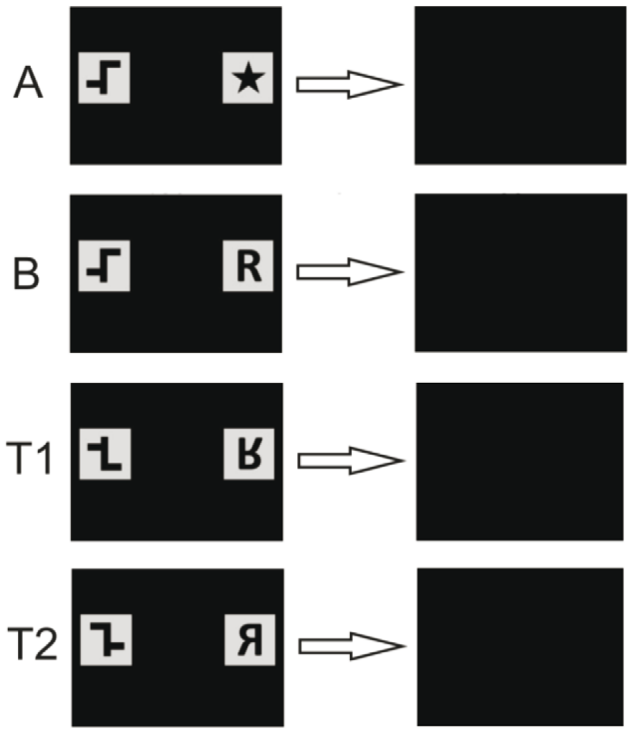
Chronology of the sequences of PowerPoint slides used in experiment 2b. The positive stimulus is shown on the left. (A) Asymmetrical shape vs. star. (B) Asymmetrical shape vs. letter ‘R’. (T1) Vertical mirror-image of stimulus pair C used for transfer tests. (T2) Horizontal mirror-image of stimulus pair C used for transfer tests. The last slide denotes the ITI.

In a first step, fish were trained in a simple two-choice discrimination task, an asymmetrical shape (positive) vs. a star (negative; Fig. 7 A). In a second step, the negative stimulus was replaced by the letter ‘R’ (Fig. 7 B). The positive stimulus remained the same. Once the individuals completed all steps successfully, the fish were accustomed to an 80 % rewarding scheme with regard to the upcoming transfer tests, as transfer test trials were unrewarded. With this scheme, the fish was only rewarded in 8 out of 10 trials, assuming a 100 % correct choice. Prior to each new session, it was determined randomly which two trials would remain unrewarded, independent of the actual choice of the fish (positive or negative). This way, fish became accustomed to the fact that sometimes there was just no reward. This precaution prevented the fish from learning that only transfer trials did not receive a food reward which otherwise could have resulted in decreased participation during these trials. To test whether the subjects could discriminate between image and mirror-image, both stimuli were reflected along their horizontal axis during the first transfer test (Fig. 7, T1). In a second transfer test, stimuli were both reflected along their vertical axis (Fig. 7, T2). Because only the ability to discriminate between reflected shapes was tested, it was necessary that the chosen stimuli, used in transfer tests, could only be matched by reflection and not by rotation. In each of the following sessions, a maximum of two transfer tests was randomly interspersed. Also during transfer tests, the positive stimulus was shown equally often on the right and on the left side. A total of twelve transfer trials for both T1 and T2 were conducted.

### Experiment 3: Visual form discrimination

**Figure 8:**
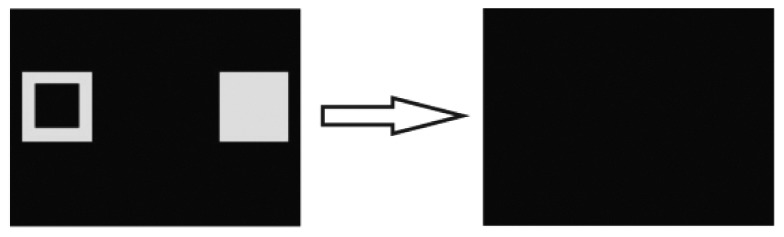
Succession of the sequence of PowerPoint slides used in experiment 3. The positive stimulus is shown on the left. The last slide denotes the ITI.

Concluding the experimental sessions, it was tested, if fish could still successfully perform in a simple two-choice visual form discrimination task. This was done to assure that neither the experimental setup, the experimenter nor the procedure itself were flawed or had impacted previous results (as most fish were unsuccessful in mastering the tasks in the second experiment), and that the obtained results represent genuine individual learning capabilities. The used stimulus pair ‘filled square’ vs. ‘blank stimulus field’ (see Fig. 8 and Table 3) had already been successfully employed in a former study [28].

### Data analysis and statistics

All graphics were constructed using Origin®Pro Version 8.0724. Average values for performance and reaction time were calculated for each individual per session/per experiment, and were then averaged across all subjects (for each experiment). Averages are given with ±1 standard deviation. Performance during transfer tests was analyzed using an interactive calculation tool for χ^2^-test of goodness of fit [84], to test whether the frequency of choices varied among reflection level of the used stimuli (horizontal or vertical mirror-image). This tool recommends a Yates' correction for continuity in tests with only one degree of freedom (df). Therefore, only the χ^2^-values as well as the p-values after Yates' correction are shown in the results section. Values of p<0.05 were considered statistically significant.

## Results

### Experiment 1a

None of the fish (n = 6) reached the learning criterion and the performance remained well below the 70 % threshold (48.9± 11 % correct choices averaged for all fish) for the whole duration of 40 sessions.

### Experiment 1b

**Figure 9:**
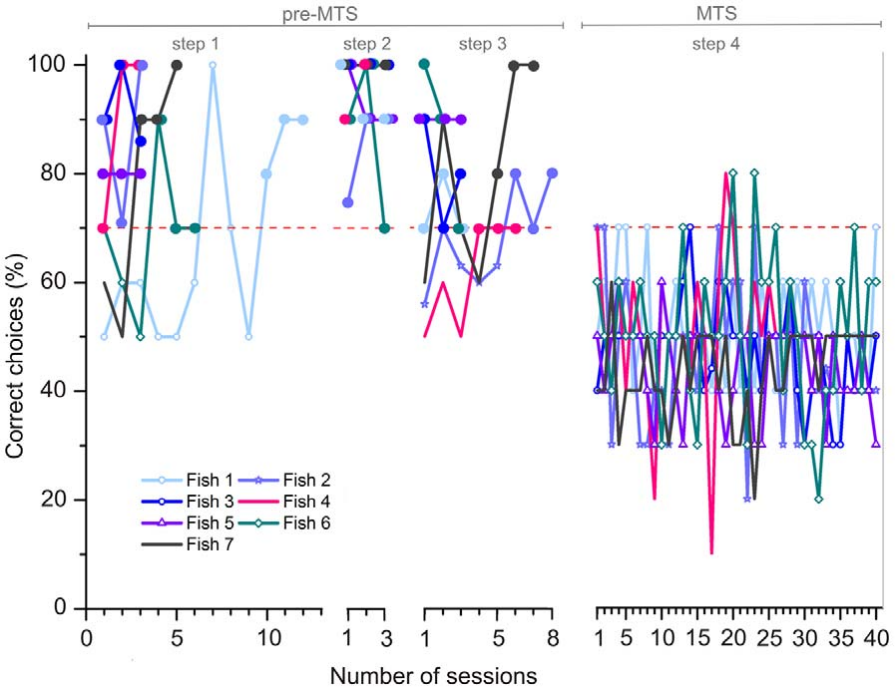
Training achievements for all individuals used in parts 1–4 of experiment 1b. The colored lines display the individual learning success for each fish, shown as the percentage of correct choices per session (à 10 trials) during pre-MTS-phase and MTS-phase. The dashed red line indicates the threshold for the learning criterion at 70 % correct choices. The three sessions necessary to reach criterion (≥70 % correct choices) are highlighted by big dots colored analogous to the individual learning curves. If two or more dots fall together at one session, they are shown overlapping each other.

All individuals (n = 7) reached the learning criterions for steps 1–3 (pre-MTS-phase) within a mean period of 5± 3.3 sessions for step 1, 3± 0 sessions for step 2, and 4.7± 2.2 sessions for step 3. Individuals showed an overall performance of 81.1± 15.2 % correct choices during the pre-MTS-phase. In contrast, no fish was able to reach the learning criterion in the subsequent MTS-phase (step 4), performance dropped down to chance level (average: 47.7± 11.3 %) for the whole period of about 40 sessions. Figure 9 shows the individual training achievements summarized for all four steps of experiment 1b. The pre-MTS-phase is shown in more detail to highlight individual differences. For completeness the MTS-phase is shown as well. Despite its complexity Figure 9 points out the important difference between the achievements during pre-MTS and MTS-phase.

### Experiment 2a

In experiment 2a, fish (n = 8) were trained to discriminate between an image and its mirror-image. There were equal numbers of horizontal and vertical image/mirror-image pairs. The number of sessions differed between 35 sessions in parts A and D, and 40 sessions in parts B and C. Fishes 1, 2, 6, and 7 participated in all four parts of the experiment (A-D). Fishes 3, 5, and 8 participated in only three parts (A-C), and Fish 9 only in part C. No fish reached the learning criterion in part A. Two fishes reached criterion in both B and C and again no fish reached the learning criterion in part D.

**Figure 10:**
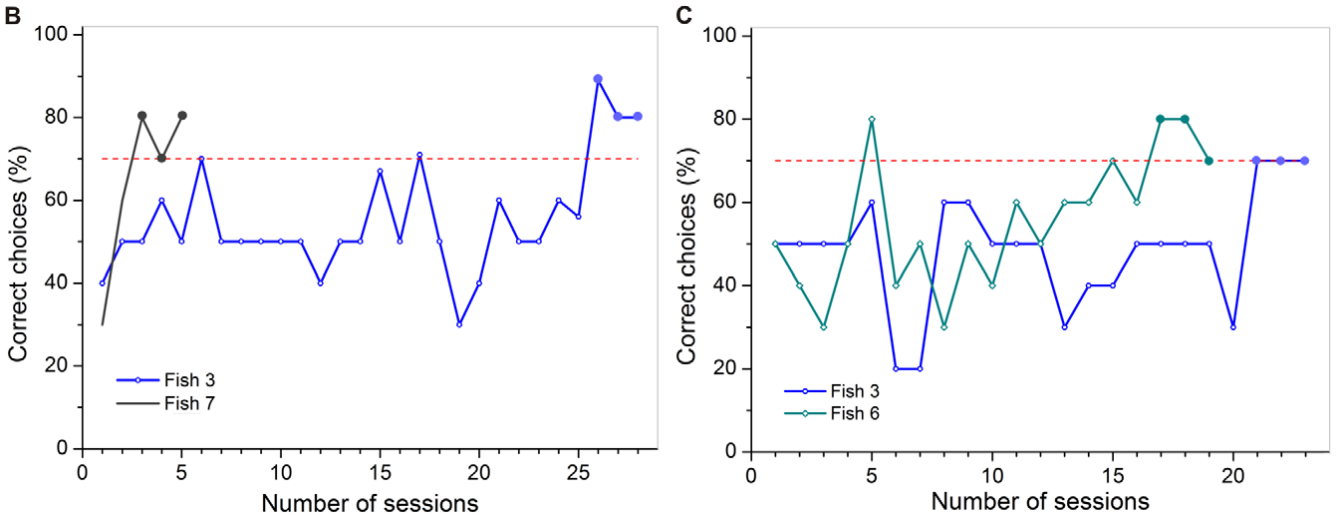
Training achievements for all individuals used in part B and C of experiment 2a. The colored lines display the individual learning success for each fish that reached criterion, shown by the percentage of correct choices per session (à 10 trials). The dashed red line indicates the threshold for the learning criterion at 70 % correct choices. The three sessions necessary to reach criterion (≥70 % correct choices) are highlighted by big dots colored analogous to the individual learning curves.

In part B, performance varied greatly. One individual reached the learning criterion within five, the other within 28 sessions (on average 16.5± 16.3 sessions, Fig. 10 B). In part C, individuals reached the criterion within a similar period of time, one after 19, the other after 23 sessions (on average 21± 2.8 sessions; Fig. 10 C).

### Experiment 2b

Only two out of seven individuals (Fishes 5 and 6) were able to master the first discrimination task and were then trained in the second task. Both individuals also reached the learning criterion in the second task but only Fish 5 participated in the following transfer tests (T1 and T2), due to its constant high performance level. Fish 6 had to be excluded from further testing after reaching criterion as it stopped being motivated to participate (would not take its reward and stayed in the rear compartment during some trials).

**Figure 11:**
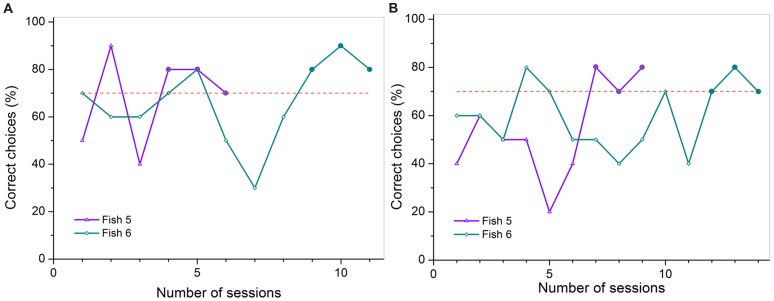
Training achievements for all individuals used in part A and B of experiment 2b. The colored lines display the individual learning success for each fish that reached criterion, shown by the percentage of correct choices per session (à 10 trials). The dashed red line indicates the threshold for the learning criterion at 70 % correct choices. The three sessions necessary to reach criterion (≥70 % correct choices) are highlighted by big dots colored analogous to the individual learning curves.

In part A of experiment 2b, Fish 5 and Fish 6 reached the learning criterion in six and 11 sessions respectively (on average 8.5± 3.5 sessions, Fig. 11 A). In part B, Fish 5 and Fish 6 were able to successfully reach the learning criterion in nine and 14 sessions (on average 11.5± 3.5 sessions, Fig. 11 B).

**Figure 12:**
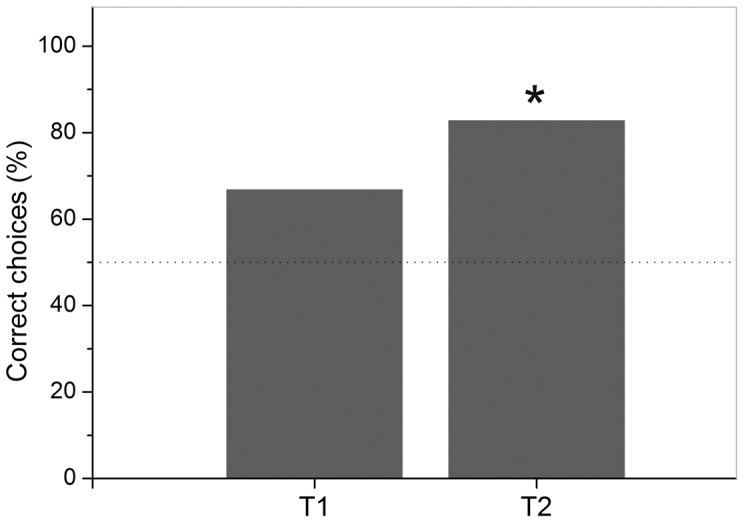
Transfer test results for Fish 5 in experiment 2b. Grey bars give the percentage of correct choices made by Fish 5 during the transfer tests T1 and T2. During each transfer, a total number of n = 12 transfer trials (no more than two per session) were inserted between the regular trials of each session in a randomized order. * =  significantly correct choices (p<0.05).

The vertical mirror-image of the stimulus pair was presented during the T1 transfer trials. Out of 12 transfer trials, Fish 5 chose eight times the correct stimulus and four times the incorrect one. This performance of 67 % correct choices during the T1 transfer trials (Fig. 12) was lower than the individual's overall mean performance level of 81.5± 14.6 % during regular trials in the same time period (T1) and did not differ significantly from chance level (n = 12, χ^2^ = 0.75, df = 1, p = 0.386). In transfer test T2, the horizontal mirror-image of the same stimulus pair as used in T1 was presented during transfer trials. Out of 12 transfer trials, Fish 5 chose ten times the correct stimulus and two times the incorrect one, resulting in 83 % correct choices (Fig. 12). Performance was comparable to regular trials (86± 8.4 %) and differed significantly from chance level (n = 12, χ^2^ = 4.083, df = 1, p = 0.043).

### Experiment 3

**Figure 13:**
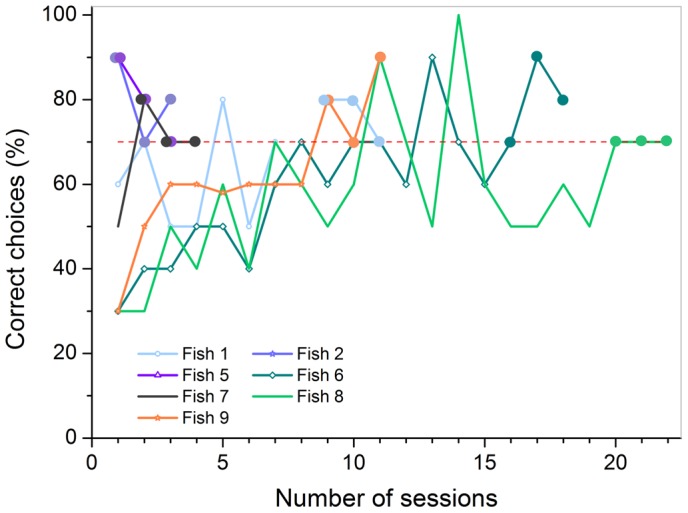
Training achievements for all individuals used in experiment 3. The colored lines display the individual learning success for each fish, shown by the percentage of correct choices per session (à 10 trials). The dashed red line indicates the threshold for the learning criterion at 70 % correct choices. The three sessions necessary to reach criterion (≥70 % correct choices) are highlighted by big dots colored analogous to the individual learning curves. If two or more dots fall together at one session, they are shown overlapping each other.

All fish (n = 7) were able to reach the learning criterion within a minimum of three sessions and a maximum of 22 sessions (on average 10.3± 7.6 sessions, Fig. 13). Fishes 2, 5, and 7 already reached criterion within the first three or four sessions. Fishes 1 and 9 both reached criterion in session 11. Fishes 6 and 8 needed comparatively more sessions to master the task and finally reached the learning criterion in sessions 18 and 22.

In conclusion, Table 5 provides an overview of the learning results for all fish in all experiments.

**Table 5:** Overview of the learning achievements summarized per individual/per experiment.

	Matching-to-sample					Two-choice-discrimination						
	Exp 1a	Exp 1b				Exp 2a				Exp 2b		Exp 3
Subject		Step 1	Step 2	Step 3	Step 4	A	B	C	D	A	B	
**Fish 1**	40	12*	3*	3*	40	35	41	40	35	40	-	11*
**Fish 2**	40	3*	3*	8*	40	35	40	40	35	40	-	3*
**Fish 3**	40	3*	3*	3*	40	35	28*	23*^+^	-	-	-	-
**Fish 4**	40	3*	3*	6*	25^+^	-	-	-	-	-	-	-
**Fish 5**	40	3*	3*	3*	40	36	40	41	-	6*	9*	3*
**Fish 6**	40	6*	3*	3*	40	35	41	19*	35	11*	14*	18*
**Fish 7**	-	5*	3*	7*	40	35	5*	40	25°	40	-	4*
**Fish 8**	-	-	-	-	-	35	40	40	-	40	-	22*
**Fish 9**	-	-	-	-	-	-	-	40	-	40	-	11*

Numbers are given for either sessions needed for criterion or sessions till the experiment was terminated. Exp =  experiment; * =  learning criterion reached after session X; ^+^ =  died after session X; ° =  terminated due to lack of motivation.

## Discussion

The present study was conducted to investigate the ability of the cichlid *Pseudotropheus* sp. to solve a MTS task (experiments 1a and 1b). It was furthermore tested whether fish could distinguish between an image and its mirror-image (experiments 2a and 2b) and between two simple visual features (experiment 3).

### Simultaneous MTS procedure (experiment 1a and 1b)

In experiment 1a, no individual reached the learning criterion in the defined time period (35–40 sessions à 10 trials). In experiment 1b, all fish reached the learning criterion in steps 1–3. In step 4, no individual reached the learning criterion in the period of 40 sessions. *Pseudotropheus* sp. may have easily learned step 1 as the task could be solved on the basis of a simple two-choice visual discrimination. The presentation of the sample stimulus at the beginning of each trial was probably ignored. As both ‘cross’ and ‘circle’ served as the positive stimulus, the fish may have learned to choose any symbol or to simply avoid the empty field to get a reward. In the same way, fish successfully could have mastered step 2. With the premise to choose just any symbol or to avoid the empty field, the introduction of the two novel stimuli would have had no remarkable effect (and indeed the performance of all fish remained above the 70 % threshold). In step 3, the sample stimulus was shown additionally in the center of the presentation. All seven individuals reached the learning criterion, potentially again either by choosing the symbol or by avoiding the empty field. In the beginning, three fish seemed to be confused by the additional stimulus in the center and performance dropped below the 70 % threshold. Nevertheless, they quickly regained criterion level. In step 4, performance dropped and fish remained unsuccessful.

In contrast to the herein presented results, Goldman and Shapiro [53] showed that goldfish (*Carassius auratus auratus*) could successfully learn to perform a simultaneous MTS procedure. In contrast to the current study, white, red, green, and blue lights were used. After the training period (70 sessions, à 120 trials), the majority of goldfish showed a performance level of 75 % correct choices, with some individuals performing above 85 %. Several goldfish performed above 70 % correct choices even before the end of the training period, i.e. before 40 sessions, whereas other individuals remained unsuccessful. The goldfish showing the quickest learning ability took about 20 sessions. Therefore, first learning results in at least some individuals could have also been expected for *Pseudotropheus* sp. during the herein used time frame. While there have been no MTS studies using two-dimensional shapes in fish, there have been a few studies testing this skill in pigeons (*Culumba livia* [85]) and bees (*Apis mellifera* [7]). In these studies, pigeons showed a performance >80 % after 30 sessions and a performance of >70 % was found in bees after 60 training trials (equivalent to one training day). These numbers indicate that some pigeons and bees reached criterion in a time frame equivalent to the number of sessions provided in the current study. Regarding the trials per session, there is some evidence that the number of trials per session does not necessarily correlate with learning success [86]. In fact, only 1 trial/day lead to better learning results than 60 trials/day in goldfish [87]. It is therefore difficult to say, whether fish in the current study could have reached the learning criterion faster if more trials per session had been performed. It is quite likely however, that at least one fish would have shown some improvement during the 40 sessions if the task could generally be solved by this species.

In the given experimental setup, *Pseudotropheus* sp. individuals were possibly neither able to obtain rules for the used set of stimuli (if ‘cross’ is the sample, then choose ‘cross’) nor were they aware of the concept of ‘sameness’ to solve the MTS tasks successfully. This would have rendered them unable to choose the correct stimulus. The initial hypothesis that *Pseudotropheus* sp. is able to solve the MTS task could not be confirmed.

### Discrimination of horizontal and vertical image/mirror-image stimulus pairs (experiment 2a)

In experiment 2a, individuals were trained to discriminate between horizontal (parts A and D) and vertical (parts B and C) image/mirror-image stimulus pairs. The initial hypothesis on mirror-image discrimination was supported by the results of this study, since three individuals successfully distinguished between F- and U-shapes and their vertical mirror-image, while no individual was able to distinguish the horizontal stimulus pairs within 35-40 sessions. These findings are consistent with results obtained from image/mirror-image discriminations in other animals, where it was shown that vertical mirror-images are generally more readily discriminated than horizontal mirror-images by octopuses [31], pigeons [36], rats [32], cats [37], rhesus monkeys [38], and humans [33], [88]–[90]. It was also shown that in general, non-mirror-image forms were better discriminated than mirror-image forms [36], [38]–[43] which might explain the low number of successful fish in the current study. However, Kirk [91] successfully trained white rats to discriminate between an ‘F’ and its horizontal mirror-image. Baboons (*Papio papio*) showed no significant difference in discrimination learning of either mirror-image pairs or asymmetric patterns [44]. Lohmann et al. [41] also found no differences between horizontal and vertical mirror-image discrimination in pigeons. Sea lions (*Zalophus californianus* [46]) and bumble bees (*Bombus impatiens* [48]) were also capable of mirror-image discriminations.

There are different opinions concerning the discriminability of mirror-images with regard to how a given stimulus is perceived by an animal and to which stimulus features are important, e.g. whether an animal attends mostly to the top/bottom/center/edge of a given stimulus [38]. For example, Riopelle et al. [38] found that rhesus monkeys restricted their attention to the bottom or top of a stimulus (see also [14]). This visual strategy would facilitate vertical mirror-image discriminations but impede discriminations of horizontal mirror-images [38]. Sutherland [31] argued that octopuses solve vertical mirror-image problems more readily because their visual system analyzes horizontal extents of a stimulus more accurately than vertical ones. A related study showed difficulties of goldfish when distinguishing between oblique rectangles (both vertical and horizontal mirror-images) compared to horizontal vs. vertical rectangles [49]. Sutherland [92] suggested that goldfish analyze stimuli according to contours and less in terms of their orientation. This would imply mirror-image confusion due to identical contours. Subsequent studies indicated that goldfish discriminate shapes by the relative numbers of points or knobs present in each shape [93], [94]. The relative number of points in an image and its mirror-image is identical and hence favor mirror-image confusion. If the same visual strategies are applied by *Pseudotropheus* sp., this could help to explain the observed difficulties in mirror-image discriminations. However, as some individuals could actually distinguish between F- and U-shapes and their vertical mirror-images it could be assumed, that those individuals used additional cues or that *Pseudotropheus* sp. uses different discrimination strategies than goldfish altogether. This would also explain the observed differences between goldfish and *Pseudotropheus* sp. in the MTS tasks. As it is often unknown which visual cues or stimuli actually matter to an animal in its natural habitat [75], [76], confident predictions for the relevance of stimuli used in laboratory experiments can hardly be made.

### Discrimination of distinct stimulus pairs and mirror-image transfer tests (experiment 2b)

In experiment 2b, seven fish were trained to discriminate between two stimulus pairs. Fish 5 and Fish 6 reached the learning criterion in both the first and the second part of the experiment. Fish 5 then performed in two mirror-image transfer tests. In the first transfer test (T1), choices did not differ significantly from chance. Fish 5 was either not capable of realizing the connection between the original stimulus pair shown during regular trials and its vertical mirror-image shown during the transfer trials, or was confused by being presented with a new experimental situation. The former seems more plausible as reaction times measured during transfer trials did not differ significantly from regular trials. As *Pseudotropheus* sp. could not transfer a learned paradigm from an image to its vertical mirror-image the two presentations must have looked distinctly different to the fish and were not recognized as being ‘the same’ or ‘the more alike’ set of symbols. During T2 transfer trials Fish 5 chose significantly often the correct stimulus. Therefore, the mirror-image must have been identified as ‘the same as’ or ‘more like’ the original stimulus pair. This would help to explain why *Pseudotropheus* sp. may have had difficulties in discriminating between horizontal mirror-images in experiment 2a. If Fish 5 had used other cues apart from the reflection to solve the task, i.e. only the negative stimulus (‘R’) contained round elements, then choices in T1 should have also been significantly often correct. The results therefore support previous research in that vertical mirror-images are discriminated more easily than horizontal mirror-images [31]–[33], [36]–[38], [88]–[90]. Surprisingly, Fish 5 did not reach the learning criterion in any of the mirror-image discrimination tasks conducted in experiment 2a.

### Visual form discrimination abilities in *Pseudotropheus* sp. (experiment 3)

All individuals (n = 7) reached the learning criterion within an average number of 10.3± 7.6 sessions. Experiment 3 was conducted to confirm that all *Pseudotropheus* sp. individuals were capable of solving a simple discrimination task and that results obtained in experiments 1 and 2 were not due to fish being sick. It served as a control to show that there were no experimental flaws and that the former obtained results represent genuine individual learning capabilities. Obviously, there seems to be a large degree of intraspecific variation, possibly indicating that such tasks or stimuli have little relevance for this species under natural conditions (resulting in only some fish mastering them). If being able to perform well in a MTS task or discriminate between images and their mirror-images was relevant under natural conditions, i.e. for survival, one could expect more individuals to learn the task at a much faster rate, as for example seen in the simple visual discrimination tasks [20], [22], [24], [28].

## Conclusion

In terms of the cognitive abilities of *Pseudotropheus* sp., the results of the present study indicate that: (1) *Pseudotropheus* sp. is capable of quick associative learning, allowing the fish to form a relationship between two events, e.g. that the appearance of a shape is connected to a reward. (2) In the given setup, *Pseudotropheus* sp. was not able to learn a simultaneous MTS within 40 sessions, which gives some indication that this species may have not been capable of rule learning in the given context. At least in the tested set up, the fish also seemed to lack a concept for ‘sameness’ which is surprising, as *Pseudotropheus* sp. was actually shown to form categorical concepts of ‘fish’ and ‘snail’ [28]. The discrepancy could be due to the relevance of the stimuli and the impact on the natural behavior of the fish. It may be specifically important for the fish to recognize and classify different organisms or objects within the habitat. (3) *Pseudotropheus* sp. can distinguish between images and their vertical mirror-images, but probably not between images and their horizontal mirror-image. There seems to be, however, a large degree of intraspecific variation, indicating that the information needed to distinguish an image from its mirror-image can generally be gained and processed by this species, but does not seem to be important for survival (otherwise, one would expect all individuals to be able to perform the skill). This is not surprising as it is rather unlikely for a fish to encounter an image and its mirror-image in a natural situation. (4) *Pseudotropheus* sp. is able to discriminate between two-dimensional geometrical stimuli. The ability to distinguish between visual stimuli aids the fish in the discrimination between friend and foe, as well as the recognition of food sources and one's territory. Being an endemic of Lake Malawi, *Pseudotropheus* sp. is highly adapted to a complex but very specific habitat [95] and may have developed skills specifically suited for this habitat.
